# Hydrogel-based autologous chondrocyte implantation leads to subjective improvement levels comparable to scaffold based autologous chondrocyte implantation

**DOI:** 10.1007/s00167-022-06886-8

**Published:** 2022-02-28

**Authors:** Thomas Richard Niethammer, Felix Uhlemann, Anja Zhang, Martin Holzgruber, Ferdinand Wagner, Peter Ernst Müller

**Affiliations:** Department of Orthopaedics and Trauma Surgery, Musculoskeletal University Center Munich (MUM), University Hospital, LMU Munich, Marchioninistraße 15, 81377 Munich, Germany

**Keywords:** Cartilage defect, ACI, Hydrogel, Scaffold

## Abstract

**Purpose:**

Scaffold-based autologous chondrocyte implantation is a well-established treatment for cartilage defects in the knee joint. Hydrogel-based autologous chondrocyte implantation using an in situ polymerizable biomaterial is a relatively new treatment option for arthroscopic cartilage defects. It is therefore important to determine if there are significant differences in the outcomes. The aim of this study is to compare the outcomes (using subjective parameters) of hydrogel-based autologous chondrocyte implantation (NOVOCART^®^ Inject) with the outcomes of scaffold based autologous chondrocyte Implantation (NOVOCART^®^ 3D) using biphasic collagen scaffold.

**Methods:**

The data of 50 patients, which were paired with 25 patients in each treatment group, was analyzed. The main parameters used for matching were gender, number of defects and localization. Both groups were compared based on Visual Analogue Scale (VAS) and subjective IKDC scores, both of which were examined pre-operatively and after 6, 12 and 24 months.

**Results:**

Significant benefits in both VAS and IKDC scores after 2 years of follow-up in both groups were found. Comparing the groups, the results showed that in the hydrogel-based autologous chondrocyte implantation group, significant changes in IKDC scores are measurable after 6 months, while it takes 12 months until they are seen in the scaffold based autologous chondrocyte group.

**Conclusion:**

Hydrogel-based autologous chondrocyte and scaffold based autologous chondrocyte show comparable improvements and significant benefits to the patients’ subjective well-being after a 2-year-follow-up.

**Level of evidence:**

III.

## Introduction

Focal cartilage defects of the knee are a common diagnosis that often leads to severe problems in quality of life and pain. The treatment of these defects is challenging and over the last decades, the recommended course of therapy has changed a lot. Since autologous chondrocyte implantation was introduced clinically in 1994 by Brittberg et al. [4], this technique has been established and according to different studies, it is by now the preferred therapy especially for full-thickness focal cartilage defects over 2.5 cm^2^ of the knee [5, 7, 11].

Over the years, autologous chondrocyte implantation (ACI) has been further developed [6]. By now, according to recent literature, especially second and third generation ACI leads to satisfying outcomes [7, 9, 15]. While collagen-based scaffold-ACI is clearly established as a good option for focal cartilage knee defects, hydrogel-based ACI (hydrogel-ACI) has been more recently developed and used as a potentially effective treatment. Reliable data on the outcomes of this procedure is not yet available, but because of the possibility of an arthroscopic implantation, promising results are expected.

For scaffold-ACI, the cultivated chondrocytes were seeded on a collagen-based membrane and implanted in an open surgery. For using a hydrogel, the cultivated chondrocytes were applied in an injectable suspension that combines a gel with a crosslinker in situ. Another difference between the two procedures is the base material. While collagen-based membranes are used commonly, there are fewer studies about hyaluronic-acid-based scaffold or hydrogel.

By now, although there are many studies on ACI, comparisons regarding outcomes of scaffold- and hydrogel-ACI in recent literature could not be found. This is probably due to the novelty of hydrogel-ACI procedure. The aim of this study is to see if there are significant differences in the subjective outcomes of these two procedures over the first 2 years after treatment. The hypothesis was that both scaffold- and hydrogel-ACI lead to good comparable results after 2 years. If hydrogel-ACI leads to comparable clinical results we recommend to use hydrogel-ACI because of its possibility to applicate it less invasive or arthroscopic.

## Materials and methods

This study was performed with an IRB (institutional review board, ID 344-12) approval from Ludwig-Maximilians-Universität München. A total of 50 patients were included consecutively in this monocentric study. The inclusion criteria were: symptomatic cartilage defects ICRS grade III-IV of the femorotibial and patellofemoral joint with a minimum defect size of 2.5 cm^2^. Exclusion criteria were: malalignment > 3–5 degrees mechanical axis deviation, osteoarthritis (Kellgren Lawrence > 2), subtotally resected meniscus in the affected compartment, rheumatoid arthritis with relevant synovitis, haemophilia-associated arthropathy and corresponding bipolar cartilage defects.

First, the scaffold-ACI group of 25 patients were treated and afterwards the hydrogel-ACI group (*n* = 25) were treated. Surgery was performed by three experienced surgeons at our clinic. After harvesting the osteochondral biopsies after 3–4 weeks, ACI surgery was performed with a knee arthrotomy in both groups. After careful debridement of the cartilage defect and measuring of the defect size scaffold-ACI (NOVOCART^®^ 3D, TETEC GmbH, Reutlingen, Germany), hydrogel-ACI (NOVOCART^®^ Inject, TETEC GmbH, Reutlingen, Germany) was injected with a syringe directly in the cartilage defect. A dual chamber syringe was used to inject a suspension of autologous cell in a solution of modified human albumin, isotonic sodium hyaluronate, human serum, and cell culture media with a cross-linker into the prepared site of the defect.

In both groups, there were 14 patients with a single defect and 11 patients with two defects that needed to be treated. Also, there were 13 men and 12 women in each of the respected treatment groups. The mean age in the hydrogel-ACI group (SD 12.8) at the time of treatment was 37.0 years, while patients treated with scaffold-ACI had a mean age of 33.9 years (SD 11.6). The mean defect size was 4.4 cm^2^ (SD 3.1) in the hydrogel-ACI group, and 5.5 cm^2^ (SD 2.8) in the scaffold-ACI group. More epidemiologic data such as etiology of the defect can be seen in Table 1.

**Table 1:** Comparison of epidemiologic data of patients in both treatment groups

	Hydrogel-ACI (*n* = 25)	Scaffold-ACI (*n* = 25)
Age at time of treatment		
Mean	37.0	33.9
Median	38.0	32.0
Min	14	13
Max	55	51
Defect size		
Mean	4.4	5.5
Median	3.0	5.0
Min	1.0	0.8
Max	12.0	12.0
Body mass index		
Mean	27.2	26.3
Median	26.3	25.6
Min	18.7	20.9
Max	39.1	35.3
Number of defects		
1	14	14
2	11	11
Localization		
Medial femoral condyle	12	9
Lateral femoral condyle	1	3
Patellar	11	12
Trochlear	1	1
Tibial	0	0
Aetiology		
Osteochondrosis dissecans	0	2
Traumatic (< 1 year)	1	3
Post-traumatic (> 1 year)	6	8
Unknown	18	11
Earlier surgical treatments		
Total	4	9
Bone marrow stimulation	4	7
Autologous chondrocyte implantation	0	1
Flake refixation	0	1
Simultaneous treatments		
Total	3	8
Osteotomy	1	0
Bone grafting	0	1
Medial patellofemoral ligament reconstruction	0	2
Collagen meniscus implantation	0	2
Anterior crucial ligament reconstruction	2	3

For match pairing, gender and number of defects as the main parameters were used. When there was more than one option, defect localization, patient age, defect size, former surgical treatments and contemporary treatments were taken in that order as further comparison parameters to find the optimal pairs.

Signed informed consent from the patients were required. The follow-up was over 24 months after treatment with both scaffold-ACI and hydrogel-ACI. All patients treated with any form of ACI are surveyed before treatment, after 6 months, 12 months and 24 months with standardized questionnaires. The two groups were compared regarding the pain level on Visual Analogue Scale (VAS) in movement and International Knee Documentation Committee (IKDC) subjective score. Both scoring systems are well established for the evaluation of knee symptoms. A complete follow-up in both tested items (VAS and IKDC score) after 2 years was given in both cases.

###  Statistical analysis

Statistical analysis and graphics were performed with IBM SPSS Statistics Version 26. For samples size calculation G*Power 3.1 was used. A total sample size of 46 were calculated. Normal distribution was tested with Kolmogorov–Smirnov and Shapiro–Wilk tests. After normal distribution was declined, group comparisons were done with Wilcoxon-test. *p*-Values smaller than 0.05 were taken as significant.

## Results

In both groups, statistical tests showed significant improvements in both VAS and IKDC scores after a follow-up of 2 years. Hydrogel-based ACI leads to an earlier improvement in the IKDC score (Table 2).

**Table 2:** Development of VAS and IKDC scores in both treatment groups

	Median	Max	Min	SD
IKDC before surgery				
Hydrogel-ACI (*n* = 25)	41.4	89.7	18.4	18.1
Scaffold-ACI (*n* = 25)	36.5	94.3	2.3	25.3
IKDC 6 months post-surgery				
Hydrogel-ACI (*n* = 25)	51.7	80.5	6.9	18.2
Scaffold-ACI (*n* = 25)	44.8	98.9	9.8	21.4
IKDC 12 months post-surgery				
Hydrogel-ACI (*n* = 25)	59.8	100.0	9.2	20.9
Scaffold-ACI (*n* = 25)	60.9	100.0	32.8	17.2
IKDC 24 months post-surgery				
Hydrogel-ACI (*n* = 25)	66.7	100.0	17.2	21.7
Scaffold-ACI (*n* = 25)	67.8	100.0	18.4	18.4
VAS before surgery				
Hydrogel-ACI (*n* = 25)	4.0	8.0	0.0	2.6
Scaffold-ACI (*n* = 25)	7.0	10.0	0.0	3.4
VAS 6 months post-surgery				
Hydrogel-ACI (*n* = 25)	3.6	9.0	0.0	2.4
Scaffold-ACI (*n* = 25)	2.6	9.0	0.0	2.7
VAS 12 months post-surgery				
Hydrogel-ACI (*n* = 25)	2.0	9.0	0.0	2.6
Scaffold-ACI (*n* = 25)	1.7	7.0	0.0	2.4
VAS 24 months post-surgery				
Hydrogel-ACI (*n* = 25)	2.0	8.0	0.0	2.3
Scaffold-ACI (*n* = 25)	2.0	8.2	0.0	2.1

**Fig. 1:**
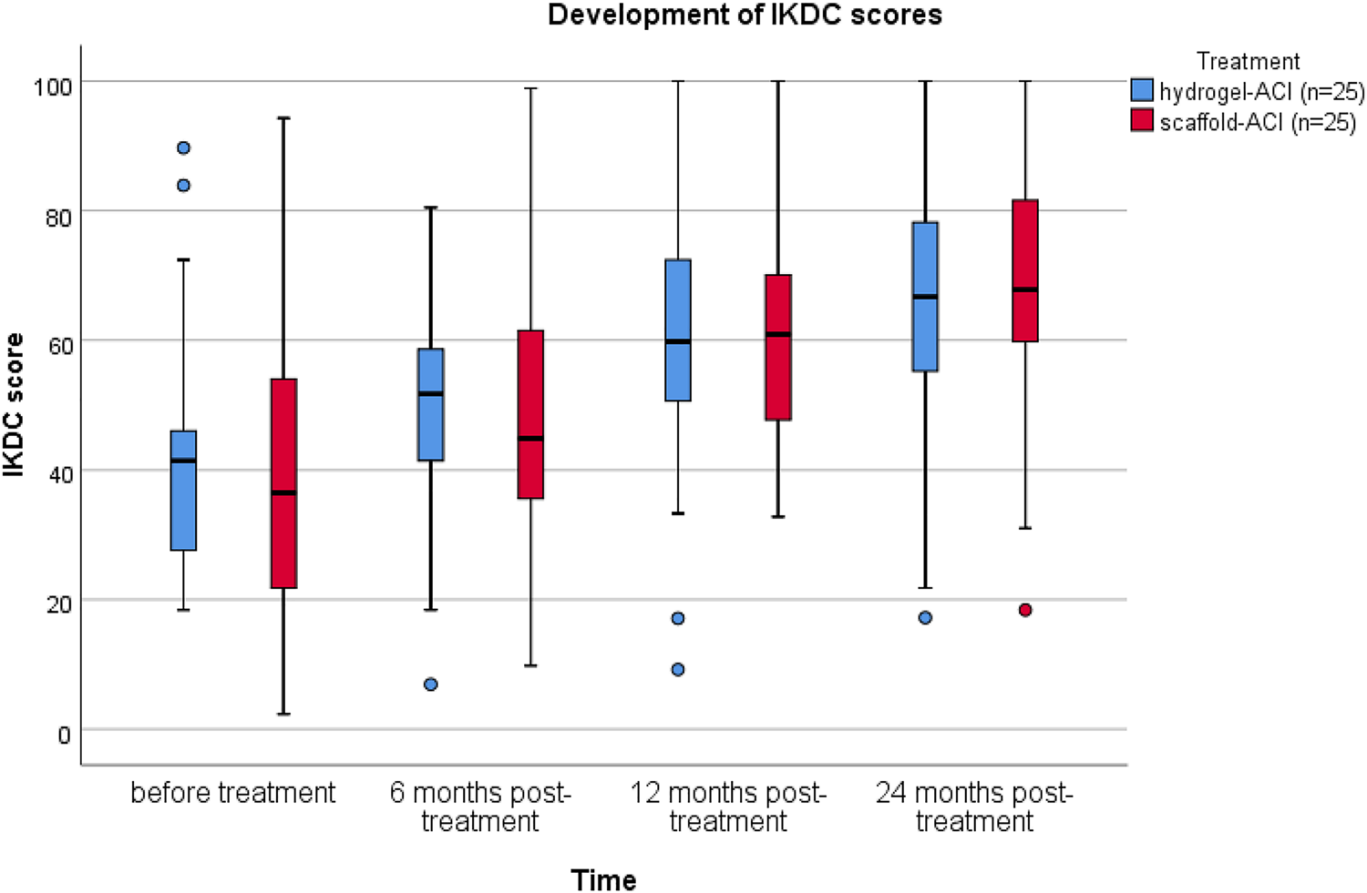
Development of IKDC score in both treatment groups. Tests have shown significant improvements in IKDC score after a follow-up of 2 years (*p* < 0.001)

**Fig. 2:**
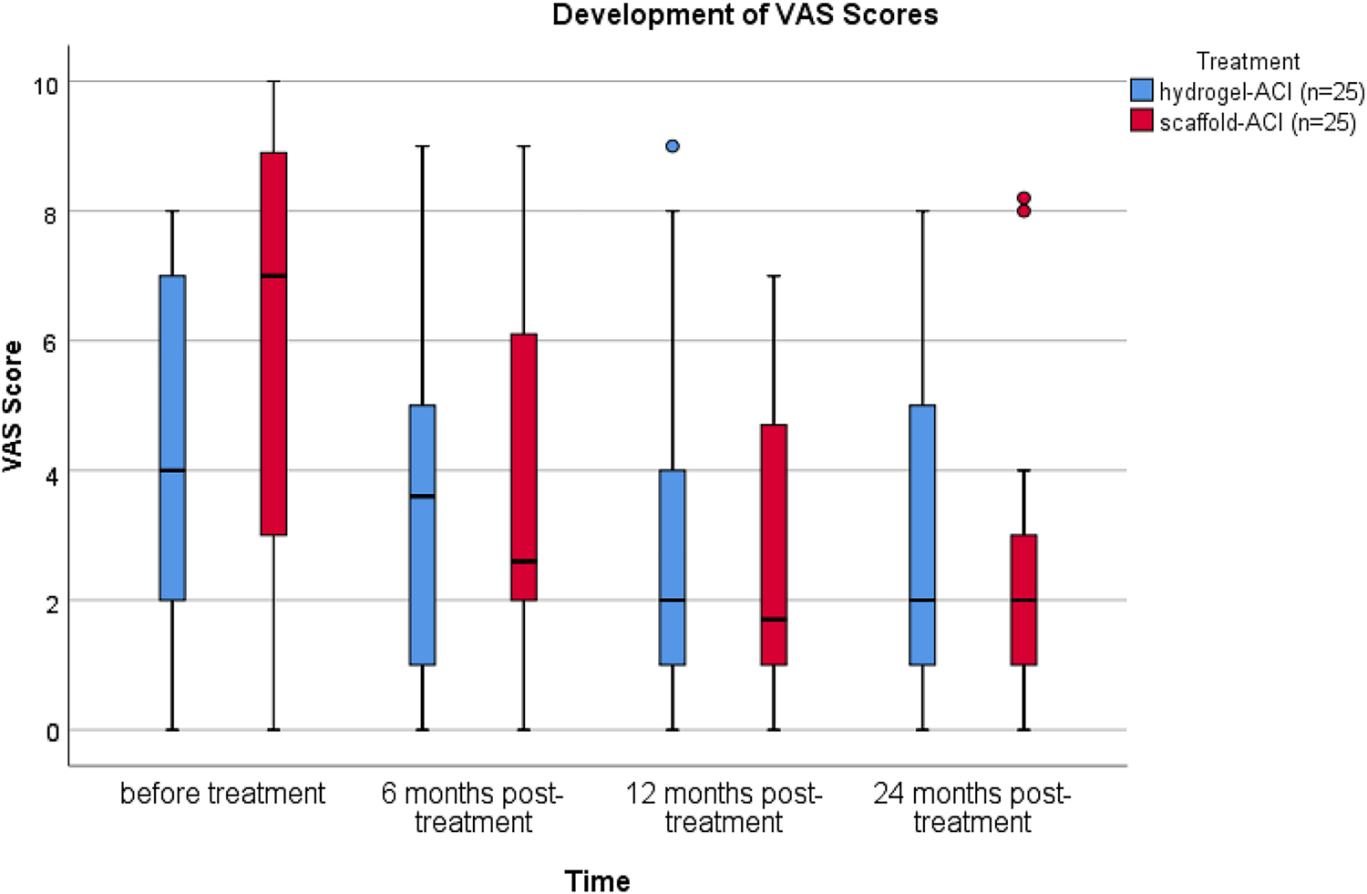
Development of VAS score in both treatment groups. Tests have shown significant improvements in VAS score after a follow-up of 2 years (*p* < 0.001)

The median IKDC score improved comparable from 41.4 to 66.7 points in the Hydrogel-ACI group and from 36.5 to 67.8 points in the scaffold-ACI group (*p* < 0.001) (Fig. 1). In the hydrogel-ACI group, the median VAS score improved from 4.0 preoperatively to a score of 2.0 after 2 years, while in the scaffold-ACI group it improved from a level of 7.0 preoperatively to 2.0 (*p* < 0.001) (Fig. 2).

**Fig. 3:**
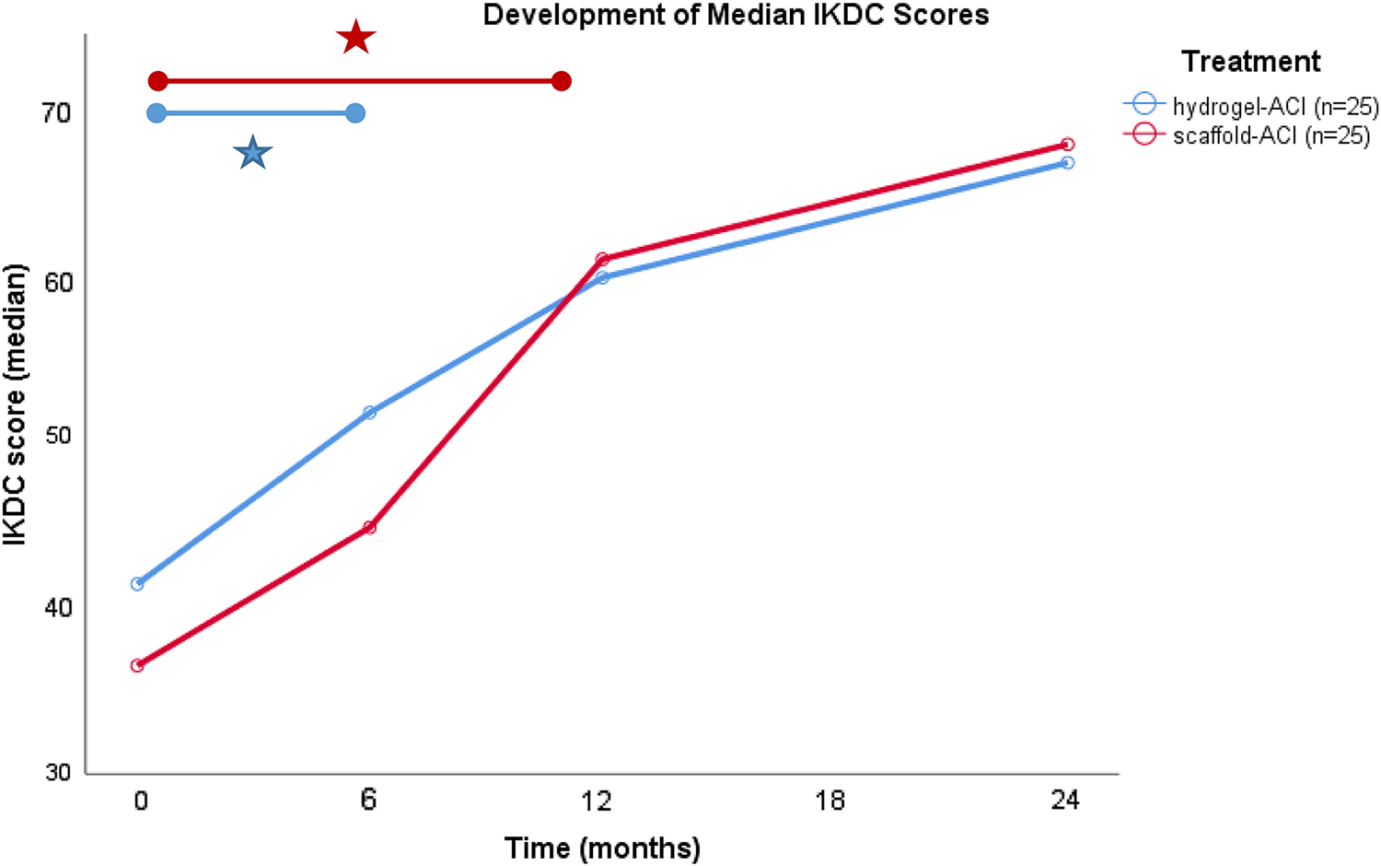
Comparison of the development of median IKDC score in both treatment groups. Significances already showed after 6 months in the Hydrogel-ACI group, while the first time significances showed in the scaffold-ACI group was after 12 months (*p* < 0.05)

**Fig. 4:**
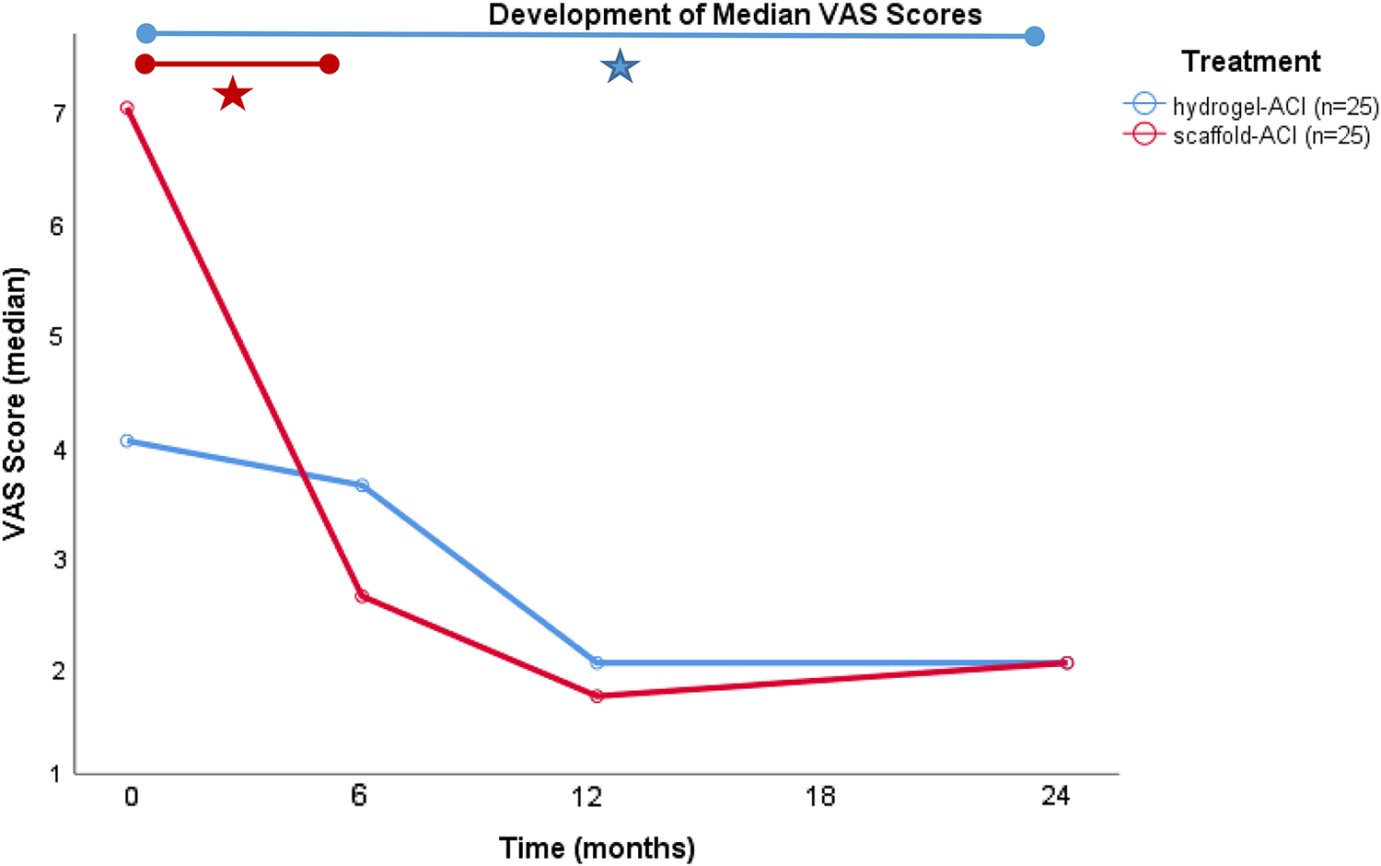
Comparison of the development of median VAS score in both treatment groups. While the improvement was significant after 6 months in the scaffold-ACI-group, the first significant improvement in the Hydrogel-ACI-group was after 24 months (*p* < 0.05)

Regarding the IKDC scores, significant improvements were found after 6 months in the hydrogel-ACI group, while the first improvements found in the scaffold-ACI group were after 12 months (*p* < 0.05) (Fig. 3). In comparison, VAS Scores showed an earlier significant improvement in the scaffold-ACI group. The same applies for the measurement after 12 months. These developments can be seen in Fig. 4.

Another finding of this study was that VAS did not improve further from 12 to 24 months. Even though there were no significant findings, the tendency in the scaffold-ACI group showed a rising median VAS score (from 1.7 to 2.0), while in the Hydrogel-ACI group, the median score stayed at 2.0 (Fig. 4).

## Discussion

The major findings of this study are that both scaffold-based ACI using a biphasic collagen scaffold and hydrogel-based ACI using an in situ polymerizable biomaterial lead to comparable subjective improvements after a follow-up of 2 years. Neither one of the two delivered significantly better results after 2 years, but there were differences in the progress over time. Hydrogel-based ACI leads to an earlier improvement in the IKDC score.

There are multiple studies on scaffold-ACI. Promising results were shown in a recent study regarding the hydrogel-ACI [3, 17, 19]. As there are several papers about first generation up to third generation ACI [1, 2, 12] and few comparisons between different generations [2, 16], there is no comparison between hydrogel-based and scaffold-based ACI.

It has been described in earlier studies that collagen-based scaffolds lead to satisfying subjective outcomes for patients when they are used in scaffold-ACI [13, 14, 18]. Similar results were found in our study. Both VAS and IKDC showed clear improvements in the comparison surveys before and after treatment. In addition, analogical results for the treatment with hyaluronic-based hydrogel were found. This suggests that neither of the two procedures is superior to the other when it comes to subjective parameters.

Regarding the scaffold-ACI, there is evidence that hyaluronic based scaffolds can lead to significant improvements. Studies showed that its use—also after a follow-up period of up to more than 10 years—delivered significant improvements and satisfying outcomes [1, 8]. Welsch et al. have compared hyaluronic based and collagen-based scaffolds in their study [21]. While the clinical results of both groups were similar and MOCART scores did not significantly vary between both groups after a follow-up of 24 months, the surface of the repair tissue was found to be in significantly better condition in the group treated with the collagen based scaffold.

Up until now, there were very few studies on hydrogel-ACI. Although there have been different reports about the clinical outcomes of hydrogel-ACI already, none of them focus on the treatment of the knee. Thier et al. as well as Krueger et al. both reported good clinical outcomes for the use in the hip joint after a follow up of one to 3 years [10, 20]. Both papers show significant improvements in subjective scores. Blanke et al. examined the short-term results of hydrogel-ACI for the knee and reported significant improvements in clinical and radiological scores 2 years after treatment [3]. Their findings were supported by the results of the present study.

In the present study, a match-paired analysis was used with two patient groups to compare patients treated with either hydrogel- or scaffold-ACI regarding their subjective outcomes after a follow-up of 2 years. Significant improvements in the first review 6 months after treatment were shown. IKDC scores seem to improve faster in the hydrogel-ACI group.

The biggest limitation of our study is the number of patients treated. A larger group of patients would lead to more conclusive results. In addition, the scores used were both subjective. Another potentially limiting factor of this study could be the novelty of the use of hyaluronic-based cell-suspensions as physicians could improve with more experience so that the quality of the outcome could also improve over the course of multiple years.

This study shows that both scaffold-ACI and hydrogel-ACI lead to comparable results at this time. Regarding the clinical scores, clinical improvements were seen earlier after the hydrogel-based ACI compared with the scaffold based ACI. Hydrogel-ACI is a less invasive procedure and can be performed all-arthroscopically, which might be the reason for the earlier clinical improvement in the IKDC score.

## Conclusion

This study showed, that both groups demonstrated a significant improvement after 2 years with neither of the two groups showing significantly better results than the other. Both scaffold based ACI and hydrogel-based ACI with a minimum incision lead to good and comparable clinical results after 2 years. Hydrogel-based ACI leads to an earlier improvement in the IKDC score.
